# Harnessing the Power of Artificial Intelligence in Cleft Lip and Palate: An In-Depth Analysis from Diagnosis to Treatment, a Comprehensive Review

**DOI:** 10.3390/children11020140

**Published:** 2024-01-23

**Authors:** Khalid A. Almoammar

**Affiliations:** Department of Pediatric Dentistry and Orthodontics, College of Dentistry, King Saud University, P.O. Box 60169, Riyadh 11545, Saudi Arabia; kalmoammar@ksu.edu.sa

**Keywords:** cleft lip, cleft lip and palate, cleft palate, artificial intelligence

## Abstract

Cleft lip and palate (CLP) is the most common craniofacial malformation, with a range of physical, psychological, and aesthetic consequences. In this comprehensive review, our main objective is to thoroughly examine the relationship between CLP anomalies and the use of artificial intelligence (AI) in children. Additionally, we aim to explore how the integration of AI technology can bring about significant advancements in the fields of diagnosis, treatment methods, and predictive outcomes. By analyzing the existing evidence, we will highlight state-of-the-art algorithms and predictive AI models that play a crucial role in achieving precise diagnosis, susceptibility assessment, and treatment planning for children with CLP anomalies. Our focus will specifically be on the efficacy of alveolar bone graft and orthodontic interventions. The findings of this review showed that deep learning (DL) models revolutionize the diagnostic process, predict susceptibility to CLP, and enhance alveolar bone grafts and orthodontic treatment. DL models surpass human capabilities in terms of precision, and AI algorithms applied to large datasets can uncover the intricate genetic and environmental factors contributing to CLP. Additionally, Machine learning aids in preoperative planning for alveolar bone grafts and provides personalized treatment plans in orthodontic treatment. In conclusion, these advancements inspire optimism for a future where AI seamlessly integrates with CLP management, augmenting its analytical capabilities.

## 1. Introduction

The progress of Artificial Intelligence (AI) has led to remarkable advancements in an array of industries, notably healthcare. Specifically, AI has had a noteworthy impact on Pediatric Care, enabling groundbreaking innovations that mitigate congenital conditions such as cleft lip and palate (CLP). Children born with a cleft lip and/or palate encounter notable obstacles. Orofacial clefts refer to a variety of conditions, such as partial or total division of the upper lip with or without division of the palate, known as cleft lip and palate (CLP) or cleft lip (CL) or identified as isolated cleft palate (CP) [1]. CL is malunion, occurring in the fourth to fifth week of the intrauterine life, where the lip, alveolus, and nasal floor have varying degrees of separation caused by the incomplete fusion of the frontonasal and maxillary processes. An incomplete cleft does not extend through all these areas. CP occurs around the eighth and twelfth weeks of intrauterine life and is caused by the failure of the palatal shelves of the maxillary processes to fuse, resulting in a separation of the hard and/or soft palates. CLP affects the lip and palate to varying degrees [2]. These characteristics may present independently, as part of a syndrome, or alongside other related abnormalities. Fortunately, the vast majority of cases of CL and CLP are non-syndromic, accounting for 70–80% of all cases. Similarly, CP is non-syndromic in approximately 50% of cases [3,4]. In addition, oral clefting manifests as a unilateral anomaly or a bilateral abnormality [5]. CLP is a prevalent abnormality worldwide. It is worth bearing in mind that cleft lip and palate conditions are prevalent among individuals of all races, albeit varying in frequency based on geographic location and ethnicity. Specifically, CL/P is more frequently observed among Asians, followed by Caucasians and, subsequently, African populations [5]. It affects approximately one in every 500–1000 live births on a global scale [6]. Overall, one out of every 700 newborns is anticipated to be born with this congenital abnormality [1]. There is a dearth of readily available data regarding the prevalence of CLP anomalies nationally in Saudi Arabia. However, retrospective analyses have uncovered various incidences, varying from 0.3% to 2.19% [7,8]. This information is vital for healthcare professionals and researchers alike, especially with approximately 355,000 children born yearly [9]. CLP is the most prevalent type of cleft deformity, followed by isolated CL, CL and isolated CP, with males exhibiting a greater incidence of clefts across all types, except for CP, which is more frequently observed in females [10]. According to a recent systematic review, a meta-analysis, the CLP prevalence was 0.45 per 10,000 live births, followed by 0.33 and 0.3 for CL and CP, respectively [11].

CLP imposes a major burden on children and their families, affecting not only feeding and speech development but also facial aesthetics and overall psychological health. Children and adults with CLP generally exhibit good psychosocial functioning, but adults may experience impaired functioning [12,13]. Children with CLP may be more susceptible to negative emotions, such as low self-esteem, a pessimistic outlook on life, and a need for assistance from others. However, their self-perception and self-worth may be influenced by their feelings toward their facial appearance. Children with CLP tend to score lower in terms of personal and social self-concept than those without CLP [14,15,16]. It is imperative to acknowledge the plausible correlation between anxiety and depression and the presence of cleft lip and palate. Individuals with CLP may be prone to experiencing these mental health disorders twice as often as those without the condition. Furthermore, discontentment with one’s facial appearance can contribute to depression in individuals with clefts [12,17]. A systematic review suggested that people with CLP may face certain psychological and social difficulties, but overall, they seem to adjust and function well. Specific psychosocial concerns have been correlated with the type of CLP anomaly. For instance, those with CLP may experience dissatisfaction with their appearance and delayed marriage, while individuals with isolated CP may have insecure attachments and learning challenges [18].

Conventional management of CLP entails several complex surgeries, rigorous speech therapy, psychological support, and frequent medical consultations—a daunting journey that does not always guarantee optimal success [19,20].

The clinical manifestations of various forms of oral clefts merit closer exploration. CL, an example of such manifestations, has the proclivity to appear either unilaterally or bilaterally, occurring on one or both sides respectively. Should it occur bilaterally, the manifestation of the cleft may be symmetrical or asymmetrical, which indicates that it may be evenly or unevenly distributed across the lip [5,21]. Within the bilateral clefts, a median proportion of the lip remains anchored in the midline to the premaxilla and the columella, containing the philtrum. In the case of complete bilateral CL, the premaxilla protrudes significantly forward, veering from the facial profile. The columella seemingly portrays a deficiency, with the alar cartilages simultaneously appearing flattened across both sides [22].

CLP can also be unilateral or bilateral and complete or incomplete. A complete unilateral CLP involves a direct connection between the oral and nasal cavities on the affected side, with the nasal septum attached to the unaffected palatal process [22,23]. An array of variations can exist in these clefts, with different levels of severity and possible combinations. Some unilateral clefts show broad separation of palatal shelves; others display less division or even an overlap. In these cases, the cleft-side palatal segment is commonly inclined inward and upward, and the vomer deviates from the midline. Similarly, bilateral clefts of lip and palate can be complete or incomplete, symmetrical or asymmetrical, relative to the equivalent affliction on both sides. The nasal chambers are directly connected to the oral cavity with bilateral clefts [5,21]. The palatal processes split equally, revealing the nasal turbinates. The premaxillary-vomerine suture, evident in cephalometric radiographs, connects the vomer and premaxilla and plays a crucial role in facial growth [2,22]. The maxilla is usually retruded in position with average height, whilst the mandible features downward and backward rotation with a significant increase in the lower facial height [24,25]. A longitudinal study conducted in Denmark found that children with CLP presented with retruded and short maxilla, with a clear increase in maxillary and nasal cavity widths. In addition, the premaxilla was protruded and deviated to the non-cleft side [26].

Isolated CP appears in the soft palate, or both hard and soft palates, but never the hard palate alone due to the fusion pattern. Depending on the severity, the cleft can range from only impinging on the uvula or soft palate to extending into the hard palate [27]. In extreme cases, the cleft can reach the nasopalatine foramen, explaining a direct communication between the nasal chambers and the oral cavity when the cleft encompasses a substantial hard palate part. The cleft’s outline can be of various forms: wide, narrow, lengthy, short, wide, narrow, pyriform, or V-shaped, and uniquely associated with mandibular retrognathia in some instances [2,25,27]. Janssen et al. (1988) concluded that children with CLP exhibit a shorter maxillary length and posterior height, with an observable prevalence of maxillary retrognathia, increased maxillary and nasal cavity width, noticeably reduced mandibular length, and mandibular retrognathia. These alterations cumulatively give rise to bimaxillary retrognathia in patients with CP [26].

The landmark longitudinal study that spanned the 1980s and 1990s, it was gleaned that uncorrected Cleft Lip and Palate (CLP) seemed to demonstrate a comparatively normal growth trajectory. However, noticeably severe proclivity, accompanied by large overjets, proclined upper incisors, and an everted major segment were persistent features. Mild contraction of the minor segment was also evident, and on rare occasions, buccal crossbites were observed [28,29].

Liao and Mars (2005) revealed that, compared with patients without CLP, those with uncorrected unilateral CLP had a significant decrease in the vertical development of the maxilla. Simultaneously, the sagittal position of the maxilla was diminished, while only minimal variations were noted in the form and positioning of the mandible [30].

Individuals with non-syndromic CLP frequently present with a spectrum of dental irregularities such as variations in tooth form, dimension, and alignment [31,32,33,34]. These irregularities have been correlated with factors such as sex, ethnic background, and the nature of the cleft condition [32,33,34]. Among the various dental anomalies, hypodontia is the most prevalent globally, followed by impacted teeth, supernumerary teeth, and microdontia [35,36,37]. In particular, hypodontia represents about two-thirds of all dental irregularities noted. A study on Saudi Arabian populations indicated that supernumerary teeth had a prevalence of 12.3%, with ectopic eruptions present in 12.5% of cases and microdontia in 45% [34]. Such dental irregularities can have profound implications, potentially compromising aesthetic appearance, the efficacy of mastication, and speech articulation [38,39].

AI-based solutions have been on the rise in many fields, including healthcare; they work by harnessing the power of big data, machine learning, and intelligent algorithms to redefine diagnosis, treatment strategies, and patient well-being. The blend of AI with the management of CLP, a revolutionary pathway emerges. This fusion sets the stage to reimagine and potentially elevate the life experience for affected children while significantly reducing the stress endured by their families. In this article, we summarize the utility of AI in cleft care among children, examining its far-reaching implications, including crafting highly tailored treatment strategies, and examine its far-reaching implications, such as improvement in recovery rates and the quality of life of children with CLP.

In this comprehensive review, our main objective is to thoroughly examine the relationship between CLP anomalies and the use of AI in children. Additionally, we aim to explore how the integration of AI technology can bring about significant advancements in the fields of diagnosis, treatment methods, and predictive outcomes. By analyzing the existing evidence, we will highlight state-of-the-art algorithms and predictive AI models that play a crucial role in achieving precise diagnosis, susceptibility assessment, and treatment planning for children with CLP anomalies. Our focus will specifically be on the efficacy of alveolar bone graft and orthodontic interventions.

## 2. Deep Learning Algorithms for Automated CLP Diagnosis

Clefts are intricate anomalies, and much effort has been made to enhance CLP diagnosis and communication among healthcare professionals [40,41]). CL can be complete or incomplete. Complete CL affects the nostrils and is commonly linked with alveolar clefting, whereas incomplete CL only results in a minor notch in the upper lip. CL may also be unilateral or bilateral. Less visible manifestations of CL include minor imperfections in the lip and ridge-like scars above the lip. In addition, less visible manifestations of clefting include minor imperfections in the lip and ridge-like scars above the lip [42]. These orofacial defects that are identified in children with CL/P are a component of a broader range of anomalies such as hemifacial microsomia, Treacher’s Collins syndrome, and Aperts syndrome [43]. The range of CP conditions spans from a submucosal cleft to complete clefting of the primary and secondary palate, as previously stated [44]. Over the years, several classification systems have been developed to categorize orofacial clefts, such as the Veau (1931), Kernahan and Stark (1958), and Nyberg et al. (1995) classifications [45,46,47]. More recently, the LAHSHAL classification, rooted in the Jensen index [26], has become the standard for surgical planning due to its precision and clarity in documenting cleft malformations. It uses a combination of letters and symbols to denote completeness and laterality while also assigning a numerical rating (1–4) to reflect severity [48].

Facial features are usually analyzed during orthodontic or maxillofacial surgery planning for diagnosis and outcome assessment. These features can provide evidence of possible pathologies, malformations, or abnormalities [49]. Orofacial clefts are typically diagnosed through physical examination and visual inspection of the infant shortly after birth [50]. Recently, the integration of AI in diagnosing and classifying orofacial clefting has shown promising results. Deep Learning (DL) has emerged as a robust tool for pattern recognition and data analysis, revolutionizing various domains, including medical imaging within Computer-Aided Diagnosis (CAD) systems. By leveraging artificial neural networks with multiple layers, DL facilitates extracting high-level features from raw data, enabling the resolution of intricate problems [51]. Convolutional Neural Networks (CNNs), a prevailing DL architecture in computer vision, emulate the human visual system to acquire and extract meaningful features from images. CNNs exhibit exceptional performance in applications such as image classification, object detection, and medical image analysis. DL-based CAD systems have transformed disease detection and diagnosis in the medical field by automating the diagnostic process [52]. These systems proficiently identify anomalies within medical images, enabling early disease detection while providing predictions of disease progression and patient prognosis [53,54,55]. In a study by McCullough et al., eight expert reviewers collected preoperative images from 800 unilateral patients with CL in the United Kingdom. These images were manually annotated for cleft-specific landmarks and rated using a previously validated severity scale. Five CNN models were trained for landmark detection and severity grade assignment. All five models demonstrated excellent performance in both landmark detection and severity grade assignment, with the residual network model performing the best (89%). The mobile device–compatible network, MobileNet, also exhibited high accuracy (86%) [56]. Agarwal and colleagues utilized artificial intelligence techniques to identify clefts using facial photographs. By combining features from a previously trained CNN and a support vector machine, they achieved high accuracy rates in identifying bilateral CLP, normal, and unilateral CLP photographs. The success rate averaged at 95%, showcasing the effectiveness of AI in cleft diagnosis [57].

Prenatal diagnosis of orofacial clefts has traditionally relied on physical examination and visual inspection of the oral cavity. However, prenatal ultrasound scans, typically performed around the 20th week of gestation, provide more accurate and comprehensive diagnostic information, enabling healthcare providers to counsel parents and make informed decisions about pregnancy [58,59]. Healthcare providers must strive to provide the most precise and comprehensive diagnostic information possible; this can help mitigate the parents’ emotional distress and facilitate informed decision-making regarding the best course of action for the pregnancy [58]. A study including 29 couples reported that 96% of the parents considered prenatal counseling as beneficial [60].

Unfortunately, however, prenatal screening may not always detect clefts, with detection rates ranging from only 9% to 50% [61]. Additionally, there is a risk of misdiagnosis, particularly when it comes to identifying cleft palate prenatally, which can lead to feeding difficulties and other abnormalities [61]. Jurek et al. (2020) developed a novel AI approach to classify ultrasound images of fetuses with cleft palate. By analyzing the sequence of bone shapes in the fetal palate, their method demonstrated a promising average efficiency of 81.6% in diagnosing cleft palate prenatally [62].

Advancements in medical imaging, particularly in the application of DL algorithms and CNNs in modalities such as panoramic radiographs, aim at improving the detection and classification of cleft anomalies [63]. Notably, Kuwada et al. developed DL models for diagnosing different types of cleft anomalies and analyzed panoramic radiographs of 383 patients with cleft alveolus (CA), both with and without CP, and 210 patients without any cleft anomalies. The overall accuracy of their second model surpassed that of the initial model and outperformed even human observers, thereby underlining the immense potential of DL algorithms in assisting healthcare professionals [64].

A recent study developed highly effective DL models for diagnosing CP in patients with unilateral or bilateral cleft alveolus based on panoramic radiographs. Two models were created to tackle this challenge. Model A used the object detection and classification functions of DetectNet to identify the upper incisor area on panoramic radiographs and classify it as either CP present or CP absent. Model B directly classified the presence or absence of CP on panoramic radiographs. The performance metrics of Model A were exceptional, with recall, precision, and F-measure all achieving a perfect score of 1.00. Models A and B demonstrated high areas under the receiver operating characteristic curve of 0.95 and 0.93, respectively, outperforming the radiologists whose scores were 0.70 and 0.63. These findings highlight the superior ability of DL models to detect and classify CP compared with that of radiologists [64,65]. It is Notably, the studies did not focus on using these models for screening purposes, and the inclusion of normal cases in the test data is necessary. Furthermore, the analysis did not delve into the differences in imaging findings between cases with and without CP [64,65]. The integration of DL-based computer-aided diagnosis (CAD) systems into clinical workflows holds immense potential in the diagnosis and assessment of CLP, providing seamless support to healthcare professionals. Please refer to Table 1 for a summary of the findings.

### 2.1. AI for Predicting the Susceptibility to CLP

The occurrence of birth abnormalities, such as CLP, is attributed to a dynamic interplay of hereditary and external influences. Several genetic components have been linked to these disorders, including mutations in Regulatory Factor 6, Msh Homeobox 1, and T-box 22 [77]. Supplementing this, comprehensive genetic studies have pinpointed correlations with areas on chromosomes 1, 2, 8, and 17 [78]. Concurrently, external factors carry weight in this context, with maternal habits such as smoking and alcohol consumption and a lack of prenatal vitamins like folic acid considered substantial risk contributors [79]. In addition the occurrence of CLP was found to be significantly associated with a positive family history of clefts, and consanguineous marriage, indicating a notable increase in the risk of CLP [80]. The application of AI to determine the likelihood of developing CLP could enhance early detection, preventive measures, and treatment plans. These sophisticated AI systems are capable of generating precise risk forecasting models by amalgamating genetic data with environmental and demographic facts. In a study by Li and co-researchers sought to unravel the complex interactions occurring among Wingless-related Integration Site (WNT) genes and their potential contribution to the development of oral clefts. They employed a trio-based design to examine the collective impact of multiple genetic variants within the WNT gene family on the risk of developing oral clefts [66]. The findings of this study indicated significant gene-gene interactions among specific Wingless-related Integration Site (WNT) genes, suggesting a complex relationship between these genes in the etiology of oral clefts and identified specific combinations of genetic variants that increased the susceptibility to oral clefts, underscoring the importance of considering interactions when studying the genetic basis of complex traits [66]. However, the authors focused only on WNT genes and included a small sample size, potentially affecting the accurate identification of gene–gene interactions. Furthermore, the genetic diversity might limit the findings’ application to other groups. Thus, further research on gene interactions in oral clefts is warranted.

The investigation by Liu et al. reveals a significant association between CLP and three cell adhesion genes (CDH1, CDH2, CTNNA2) in Chinese case-parent trios [67]. The study involved 1475 CLP case-parent trios and 1962 controls and analyzed 1455 single-nucleotide variation (SNV) in 158 candidate cell adhesion genes. A machine learning algorithm was used to investigate both two-way and multi-way interactions that may affect the risk of CLP. The findings reveal significant gene-gene interactions among three genes (CDH1, CDH2, and CTNNA2) involved in cell adhesion pathways, indicating their association with the risk of non-syndromic CLP in the Chinese population [67]. This result highlights the importance of understanding gene-gene interactions in defining CLP’s genetic underpinnings. However, the modest sample size may limit statistical strength, and application to diverse ethnicities requires caution. The research’s exclusive focus on cell adhesion genes also mandates contemplation of genetic and environmental factors. Future studies should evaluate gene-gene interaction functionality and the precise role of cell adhesion genes in CLP’s genesis [67]. Please refer to Table 1 for a summary of the findings.

### 2.2. AI in Alveolar Bone Defect Grafting: Advancements and Applications

Secondary alveolar bone grafting is the preferred approach for addressing alveolar clefts, particularly when the canine root is nearing completion. This procedure aims to restore the structural integrity and functionality of the alveolar ridge, creating a solid foundation for the proper eruption of permanent teeth and the formation of a stable dental arch, as well as promoting optimal dental and facial development [81,82]. It coincides with the accelerated eruption of teeth and the completion of midfacial growth and development, typically observed in children aged 9–12 years [81,82]. This reconstruction enables orthodontic tooth movement, which plays a pivotal role in comprehensive maxillary arch development, repair of oronasal fistulas, and upper lip and nose support [81,82]. The most optimal source of autogenous bone for alveolar cleft reconstruction is an autogenous bone graft harvested from the anterior or posterior iliac crest, commonly known as the gold standard bone graft [83].

The success of secondary alveolar bone grafting is influenced by various factors, including dental development, age, cleft size, and the timing of orthodontic treatment [84,85], as well as the timing of the graft (i.e., performed before the eruption of maxillary permanent canines), choice of surgical materials, and use of presurgical orthodontics [85]. However, there is insufficient evidence to determine whether the width or volume of the cleft has an impact on the clinical outcomes of the graft [85]. Cone beam computed tomography (CBCT) offers a three-dimensional (3D) assessment of alveolar bone defects in patients with CLP [86,87], enabling more precise surgical planning and thus minimizing surgical complications [88]. Nevertheless, such an analysis is complex, costly, and time-consuming.

To address these challenges, AI has been increasingly used in 3D image analysis tools [68,69]. The integration of AI technology aims to simplify and enhance the analysis process, ultimately streamlining the assessment of 3D images for alveolar bone defects. Zhang et al., 2020 investigated a novel approach to improve the accuracy of volume estimation in alveolar cleft grafting procedures [68,69]. The study used a 3D U-Net model and parameterized the non-linear mapping from the one-channel intensity CBCT image to six-channel inverse deformation vector fields (DVF). The results indicated that the proposed method achieved an average accuracy as high as 92% in estimating graft volumes, suggesting that volumetric registration techniques can provide valuable data for surgical planning, treatment evaluation, and postoperative outcomes assessment [68,69]. However, the accuracy of the volumetric registration techniques may vary depending on factors such as image quality and the choice of specific registration algorithms. Notably, the scope of this study was limited to a specific type of grafting procedure, and its findings may not be universally applicable to all surgical techniques. Wang et al. introduced a groundbreaking advancement in evaluating unilateral CP [68,69]. Leveraging DL algorithms and CBCT produces unparalleled accuracy and automation in the 3D segmentation of maxillae and associated defects. With an impressive average segmentation accuracy of 96%, surpassing manual methods, this breakthrough enables comprehensive morphometric quantification, empowering clinicians and researchers with detailed analysis and visualization of CP conditions [68,69]. They found that significant hypoplasia of the maxilla on the cleft side existed mainly in the pyriform aperture and alveolar crest area near the defect. Automation significantly reduces time and effort, streamlining treatment planning and monitoring processes for improved patient care. While recognizing limitations such as image quality, patient age, and complexity of CP deformity, this new approach sets a new standard in computer-assisted analysis, fostering advancements in the field and promising enhanced surgical treatment planning and outcome evaluation for unilateral CP patients [68,69]. Please refer to Table 1 for a summary of the findings.

### 2.3. AI-Enhanced Orthodontics for Adolescent CLP Patients

Orthodontic intervention is pivotal in the integrated care of patients with CLP. The advent of a secondary alveolar bone graft has made orthodontic treatment a standard procedure in CLP management. These treatments help methodically align teeth, correct anterior and posterior crossbite, and rectify existing malocclusions [2,22].

Orthodontic intervention in children with CLP primarily involves comprehensive fixed orthodontic appliances that focus on the upper arch. This approach is instrumental in the strategic retraction of impacted canines and in aligning dentition. As the child’s craniofacial development reaches completion, a comprehensive orthognathic strategy is adopted to meticulously correct the overall malocclusion and facial esthetics [85,89]. In children with mild Class III malocclusion, a nuanced camouflage method may be used. Tailored particularly for milder manifestations, this approach may selectively use extractions to mitigate dental crowding, rectify the midline, and culminate in a well-balanced occlusal relationship [21,89].

As a supplementary measure in the mixed dentition stage, protraction headgear can be used to counteract maxillary retrusion. This device smoothly coaxes the maxillary dentition forward, improving facial harmony and occlusal function. Each therapeutic step is taken with a singular goal—achieving a functional, esthetically pleasing dental arrangement that harmonizes with the patient’s overall facial structure [2,22]. Despite this, to date, AI has not been exploited in the clinical decision-making process for selecting growth modification strategies nor for forecasting the effectiveness of protraction headgear in treating midfacial hypoplasia in children afflicted with CLP anomalies.

Since orthodontic treatment amongst patients with CLP may involve the extraction of teeth to attain the treatment goals. In recent research, several mathematical models and AI techniques have been developed and tested to accurately assess the need for extractions and determine optimal extraction patterns in orthodontic cases. Let us delve into some of these studies and their findings. A study conducted by Takada [70] and Yagi [71] developed a mathematical model to assess the need for extractions and determine the optimal extraction pattern for orthodontic cases. The model used standardized photographs, radiographs, and orthodontic casts as input data. It compared the features of the presenting malocclusion with preexisting templates in the system, making multiple decisions based on case traits. The model’s accuracy was evaluated against clinicians’ decisions, yielding a success rate of 90.4%. Overjet and upper and lower arch length discrepancies were identified as the key factors influencing extraction decisions [70]. There was 86% agreement between the extraction decision made by the orthodontist and those recommended by the AI model [71]. Subsequently, Xie and colleagues (2010) incorporated artificial neural networks (ANN) into the model and achieved an 80% accuracy rate for differentiating between extraction and non-extraction cases among patients aged 11–15 years [72]. Jung and Kim (2016) used the R programming language to develop a model that effectively identified patients requiring extraction. Compared with the treatment plans of an experienced orthodontist, the model demonstrated an identification accuracy of 93% for patients in need of extractions, with an overall accuracy of 84% for the extraction plan [73]. Li and colleagues (2019) reported that compared with k-nearest neighbors, ANN achieved an accuracy of 94% in predicting the need for extraction, with an anchorage pattern accuracy of approximately 92.8%. The curves of Spee, angle ANB, and upper arch crowding were identified as the most influential features for accurate prediction [74].

Children with CLP may require an orthognathic approach after craniofacial growth cessation to address skeletal discrepancies and achieve a harmonious face. In a South Korean study, Choi et al. (2019) used a sophisticated neural network-based AI framework for analyzing and planning orthognathic surgical procedures [75]. Notably, this model included a specialized DL algorithm adept at assessing facial structures and identifying defects; its development driven by extensive training with a diverse compilation of facial scans, outcomes of surgeries, and postsurgical recovery data; and its capacity to significantly improve the speed and accuracy of the planning of complex operations. In this investigation, 316 patients were stratified into those undergoing surgery (n = 160) and those undergoing non-surgical treatment (n = 156). Diagnostic accuracy for discerning the suitability of surgery versus non-surgical methods achieved remarkable rates, registering a 95% success rate within the training cohort, 97% in the validation cohort, 96% upon evaluation in the test cohort, and an overall success rate of 96%. Notably, the model was 100% successful in identifying the necessity for surgical procedures in Class II and III malocclusions across all cohorts. Additionally, for those categorized under Class II surgical interventions, the model skillfully differentiated between cases requiring tooth extractions and those that did not, achieving success rates of 95% in the control group and 100% in the experimental group with an overall accuracy of 97% [75]. Nevertheless, the authors identified a limitation of the AI framework: its dependency on the scope and breadth of the training data. The representativeness of different populations within the dataset is crucial; any shortfall, particularly with rarer conditions or less represented demographic groups, could negatively influence the model’s effectiveness and generalizability [75]. Shin et al. (2021) also used a DL system to assess the demand for orthognathic surgery for skeletal malocclusion by examining both transverse and longitudinal cephalograms in 840 Korean individuals (244 with Class II malocclusion, 447 with Class III, and 149 with facial asymmetry). Each patient was a candidate for orthognathic surgery to correct dentofacial deformities, as well as Class II and III malocclusions. Among the 413 cases included in the testing phase, the model successfully categorized 394 cases (Shin et al., 2021), corresponding to an accuracy of 95.4%, sensitivity of 84.4%, and specificity as high as 99.3%. As a potential diagnostic aid, the innovation promises to refine the accuracy of medical assessments and facilitate personalized surgical strategies by factoring in individual cranial measurements [76]. The study’s primary limitation is its concentration on a single ethnic group, which potentially limits the model’s generalizability to a broader populace. Future studies need to diversify the model’s dataset to extend the algorithm’s precision and usefulness across different ethnic backgrounds. Please refer to Table 1 for a summary of the findings.

### 2.4. Limitations and Future Directions

The burgeoning integration of AI in CLP care including orthodontics presents promising advancements yet is not devoid of limitations. Few high-quality, large-scale datasets of patients with CLP are available. AI algorithms require diverse and representative datasets for training and validation, yet few such datasets are available [90,91], hampering the development and generalizability of AI models in CLP care. Furthermore, training AI models often inherit biases present in the input data, potentially leading to skewed clinical recommendations [91]. To mitigate this, data from a varied patient demographic is essential. However, a lack of standardization in radiographic protocols and annotations, making it difficult to train AI systems effectively [92]. Most AI applications in orthodontics for patients with CLP are in their infancy, focusing on diagnosis and treatment planning. However, continual assessment of treatment progress and automated adjustments are areas ripe for innovation. Moreover, AI’s role in predicting treatment outcomes, particularly in the context of growth modification therapy such as protraction headgear, remains underexplored. Real-time feedback is crucial in orthodontic treatment monitoring. Although AI can automate the monitoring process, current systems often cannot provide real-time feedback and guidance to clinicians. This limitation restricts the utility of AI in CLP management. The use of AI in CLP care also raises ethical and legal concerns. Patient privacy, data security, and liability issues must be carefully addressed to ensure compliance with regulatory frameworks and to maintain patient confidentiality. Establishing comprehensive ethical and legal frameworks is essential for the successful integration of AI into healthcare [92]. AI-driven systems also face the hurdle of gaining trust from practitioners and patients alike. The impersonal nature of AI-driven decisions and the lack of transparency in algorithmic processes can cause reluctance to adopt these technologies. Therefore, future AI solutions must incorporate explicability features that demystify decision-making processes for clinicians. Additionally, AI systems must operate within an ethical framework that prioritizes patient welfare and consent [92].

Despite these challenges, advancements in machine learning algorithms and computational power imply that AI has a significant trajectory of growth and could potentially revolutionize CLP care in orthodontics. Integrating AI tools that provide real-time diagnostic and therapeutic feedback during procedures can enhance precision and outcomes. Future studies should create federated learning systems that allow AI models to learn from decentralized datasets without direct data sharing, thus preserving privacy. Developing robust, standardized protocols for data collection and annotation will also be critical in advancing AI systems that are both effective and generalizable. Concurrently, there must be a concerted effort to establish rigorous validation frameworks that ensure the reliability and safety of AI applications in clinical practice [90]. Ultimately, the cooperative efforts between AI researchers, data scientists, clinicians, and patients will forge AI tools that not only refine care for individuals with CLP but also uphold ethical considerations and foster trust within the healthcare community.

In conclusion, it is important to recognize that this review article may be susceptible to the biases commonly associated with comprehensive reviews. The present study focuses on specific aspects of CLP anomaly and analyzes a limited number of articles. It is crucial to note that further extensive clinical trials and rigorous systematic reviews are needed to fully explore the intersection of AI and CLP care in various aspects of CLP and advanced technology.

## 3. Conclusions

In this review, we aimed to investigate the transformative potential of AI in the management of children with CLP anomalies. Our analysis focused on the utilization of DL models in the diagnostic process and the prediction of susceptibility of CLP, which at times surpassed human capabilities in terms of precision. Additionally, ML algorithms can enhance preoperative planning for alveolar bone grafts, and aid in orthodontic treatment can provide personalized treatment plans, optimize the orthodontic process, and yield enhanced results. While there is still much to explore in this field, the advancements made thus far inspire optimism for a future where AI seamlessly integrates with CLP management, augmenting its analytical capabilities.

## Figures and Tables

**Table 1:** Characteristics of the reviewed studies.

Author	Objective	Sample	AI Methods	Findings
McCullough et al., 2021 [56]	Diagnosis and severity assessment	Eight experts reviewed 800 preoperative images from unilateral patients with CL in the United Kingdom. These images were manually annotated for cleft-specific landmarks and rated using a previously validated severity scale.	Five CNN models were trained for landmark detection and severity grade assignment	All five models demonstrated excellent performance in both landmark detection and severity grade assignment, with the residual network model performing the best (89%). The mobile device–compatible network, MobileNet, exhibiting high accuracy (86%).
Agarwal et al., 2018 [57]	Diagnosis and severity assessment	Photographs of 136 bilateral CLP, 670 normal cases, and 412 unilateral CLP.	CNN, specifically AlexNet model and SVM classifier Radial Basis Function	The success rate averaged at 95%. The accuracy for UCLP and BCLP were 92% and normal cases at 99%.
Jurek et al., 2020 [62]	Pre-natal Diagnosis	49 histograms, 13 of which are diagnosed with CLP, and 36 are normal at 11–13 weeks of gestation.	DL algorithm, applying a parser for a GDPLL(*k*) string grammar (Generalized Dynamically Programmed LL(*k*) grammar) for classifying abnormalities (in the recognition phase)	The average efficiency was 81.6% in diagnosing CP prenatally, with 40 out of 49 correctly diagnosed (29 normal cases and 11 cases with CLP.
Kuwada et al., 2021 [64]	Diagnosis	Panoramic radiographs of 383 patients with CA, both with and without CP, and 210 patients without any cleft anomalies.	Two DL models on the DetectNet	The overall accuracy of their second model surpassed that of the initial model and outperformed even human observers, thereby underlining the immense potential of DL algorithms in assisting healthcare professionals. Model 1 had a false positive detection accuracy in 12/30, while model 2 had reduced false positive detection in 1/30.
Kuwada et al., 2021 [64]	Diagnosis	Panoramic radiographs of 491 patients with unilateral or bilateral CA.	Two DL models on the DetectNet	Models A and B demonstrated high areas under the receiver operating characteristic curve of 0.95 and 0.93, respectively, outperforming the radiologists whose scores were 0.70 and 0.63.
Li et al., 2015 [66]	Gene interactions	A trio-based design to examine the collective impact of multiple genetic variants within the WNT gene family on the risk of developing oral clefts.	G × G interactions using machine learning and regression-based methods	Significant gene-gene interactions among specific WNT genes, suggesting a complex relationship between these genes in the etiology of oral clefts and identified specific combinations of genetic variants that increased the susceptibility to oral clefts
Liu et al., 2019 [67]	Gene interactions	1475 CLP case-parent trios and 1962 controls and analyzed 1455 single-nucleotide variation (SNV) in 158 candidate cell adhesion genes.	A machine learning algorithm was used to investigate both two-way and multi-way interactions that may affect the risk of CLP	The findings reveal significant gene-gene interactions among three genes (CDH1, CDH2, and CTNNA2) involved in cell adhesion pathways.
Zhang et al., 2020 [68]	Diagnosis and alveolar graft assessment	The dataset consists of 21 CBCT images of unilateral and bilateral CLP patients to undergo the secondary alveolar cleft grafting surgery.	A 3D U-Net model and parameterized the non-linear mapping from the one-channel intensity CBCT image to six-channel inverse deformation vector fields (DVF).	The average accuracy was as high as 92% in estimating graft volumes
Wang et al., 2021 [69]	Diagnosis and maxillary defects	CBCT images of 60 patients with unilateral CP were acquired.	DL algorithms to assess the maxilla and its defects.	The success rate averaged segmentation accuracy of 96%, surpassing manual methods.
Takada et al., 2009 [70]	Orthodontic extraction decision	188 conventional orthodontic records of patients with good treatment outcomes were collected.	A mathematical model	The model’s accuracy was 90.4%. Overjet and upper and lower arch length discrepancies were identified as the key factors influencing extraction decisions.
Yagi et al., 2009 [71]	Orthodontic extraction decision	193 females who underwent orthodontic tooth-extraction treatment that was considered successful.	A mathematical model	The model’s accuracy was 86%. Overjet and upper incisor protrusion were identified as the key factors influencing extraction decisions.
Xie et al., 2010 [72]	Orthodontic extraction decision	200 patients; among them, 120 were accepted for extraction treatments, and 80 were chosen for non-extraction treatments.	DL algorithms using ANN	80% accuracy rate was achieved for differentiating between extraction and non-extraction decisions.
Jung and Kim, 2016 [73]	Orthodontic extraction decision	156 patients with 12 cephalometric variables and 6 indexes.	Four neural network machine learning models	93% identification accuracy for patients in need of extractions, with an overall accuracy of 84% for the extraction plan.
Li et al., 2019 [74]	Orthodontic extraction decision	302 cases from the Department of Orthodontics, West China Hospital of Stomatology.	DL algorithms using ANN	An accuracy of 94% in predicting the need for extraction, with an anchorage pattern accuracy of approximately 92.8%. The curves of Spee, angle ANB, and upper arch crowding were important features for accurate prediction.
Choi et al., 2019 [75]	Orthognathic surgery	316 cases of Korean patients who visited the Department of Orthodontics, Seoul National University Dental Hospital.	DL algorithms using ANN	97% accuracy for discerning the suitability of surgery versus non-surgical methods. Achieved 95% in the control group and 100% in the experimental group, with an overall accuracy of 97% in identifying cases requiring extractions.
Shin et al., 2021 [76]	Orthognathic surgery	840 Korean patients (244 with Class II malocclusion, 447 with Class III, and 149 with facial asymmetry).	DL algorithms using CNN	An accuracy of 95.4%, sensitivity of 84.4%, and a specificity as high as 99.3% was achieved in determining the need for orthognathic surgery.

## Data Availability

Not applicable.
